# Physiological and biochemical characteristics of milk thistle (*Silybum marianum* (L.) Gaertn) as affected by some plant growth regulators

**DOI:** 10.1002/fsn3.4233

**Published:** 2024-06-11

**Authors:** Sahar Fanai, Davood Bakhshi, Bohloul Abbaszadeh

**Affiliations:** ^1^ Department of Horticultural Science University Campus 2, University of Guilan Rasht Iran; ^2^ Faculty of Agricultural Sciences, Department of Horticultural Science University of Guilan Rasht Iran; ^3^ Department of Research Center on Cultivation & Domestication of Medicinal Plants Agricultural Research Education and Extension Organization (AREEO) Karaj Iran

**Keywords:** Brassinosteroid, drought, Silymarin, spermidine

## Abstract

Milk thistle (*Silybum marianum* (L.) Gaertn) is a globally and widely used medicinal plant that contains silymarin. This plant has antioxidant, antimicrobial, anticancer, hepatoprotective, cardiovascular‐protective, and neuroprotective effects. Plant quality, yield, and phytochemicals, especially silymarin content, change under various conditions like drought stress. Therefore, this research studied plant growth regulators (PGRs) like salicylic acid, spermidine, and brassinosteroid to increase plant tolerance to drought stress. Experimental treatments include different levels of irrigation (25%, 50%, 75%, and 90% field capacity), and foliar spraying including salicylic acid (75 and 150 mg/L), spermine (70 and 140 mg/L), and brassinosteroid (1 and 1.2 μM), separately, and water as a control and a secondary factor. The results revealed that the highest amount of leaf phenolic compounds was observed in the highest drought stress level (25%) and 75 mg/L salicylic acid application. Furthermore, brassinosteroid at different concentrations and salicylic acid (75 mg/L) increased leaf flavonoid content compared to other treatments. In 50% field capacity, foliar application of salicylic acid (150 mg/L) significantly increased seed yield by approximately 75% compared to control under the same stress level. Brassinosteroid application (1 μM) under 75% field capacity significantly increased the seed's taxifolin amount by 159%. Additionally, salicylic acid noticeably increased the silychristin concentration. The concentration of silydianin in the seed has also been increased under drought stress and foliar spraying with PGRs. Compared to the control, using spermidine below 75% field capacity caused an increase in its concentrations by over seven times. The highest silybin A amount was obtained in 50% field capacity and foliar150 mg/L salicylic acid. Taxifolin, silychristin, silydianin, silybinin B, iso‐silybinin A, and iso‐silybinin B compounds were identified in the seed extract. Generally, foliar spraying using plant growth regulators increased the number of silymarin compounds under drought stress conditions and field cultivation conditions.

## Graphical abstract

A chromatogram diagram of HPLC related to the main phenolic compounds in seed extract under drought stress was shown. According to the results, the maximum of taxifolin with foliar application of brassinosteroid (1 μM) under 75% field capacity. Most silydianin was obtained by the application of spermidine in 75% field capacity. Silybin A has a maximum amount at 50% field capacity and 150 mg/L salicylic acid.

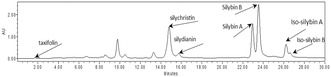

## 1 INTRODUCTION

*Silybum marianum* (L.) Gaertn is an annual or biennial herbaceous plant with a height of 100–200 cm and its habitat is in the Mediterranean regions. It has spear‐shaped leaves with 15–60 cm long and thorny edges, and the flowers of the plant are 4–12 cm long, red‐purple in color and belong to the Asteraceae family (Karkanis et al., 2011; Karimzedah et al., 2001; Gresta et al., 2007). It is cultivated in different parts of the world and is in the group of widely used medicinal plants (Boulos, 2000; Karkanis et al., 2011). Different parts of the plant, including the fruit, dry pericarp, and its seeds, have compounds that have antioxidant, antifungal, and immune‐modulating properties (Andrzejewska et al., 2011; Tayoub et al., 2018). Milk thistle seeds contain an isomeric mixture of flavonolignans known as silymarin (Morazzoni & Bombardelli, 1995). Silymarin is extracted from the extract of the fruits of this plant and is a complex composition of six flavonolignans as well as flavonoids (Biedermann et al., 2014; Giuliani et al., 2018). Silybinin, isosilybinin, silychristin, and silydianin are the main components of silymarin (Burgess, 2003). Other compounds include silybinin A, silybinin B, isosilybin A, and isosilybin B as diastereoisomers, and taxifolin is also present in it (Kvasnička et al., 2003). The yield and quality of milk thistle, for example, the content of silymarin in the plant is related to the environmental conditions of plant cultivation, soil conditions, density of plant cultivation, harvesting stage, and plant genotype (Karkanis et al., 2011; Katar et al., 2013). Because the exposure of the plant to drought stress affects the production of secondary metabolites (Belitz & Sams, 2007), water relations of the plant, the photosynthesis of leaves, the absorption of nutrients, and also the transfer of assimilates (Egilla et al., 2005).

In a specific study, it was determined that plant density has a significant impact on seed yield and plant growth. Increasing plant density leads to a considerable increase in seed yield and plant biomass in the first year of the research. However, in the second year, plant density reduced the content of silymarin in the seeds, and foliar application of plants with mepiquat chloride had no significant effect on the studied parameters. In addition, the application of drought stress during the period from March to May has been shown to result in a decrease in silymarin content and seed yield (Arampatzis et al., 2019).

Research has shown that milk thistle seed yield is severely affected and reduced under drought stress conditions (Zangani et al., 2018). One of the plant approaches to drought tolerance is the use of plant growth regulators (PGRs) (Kumari, Avtar, et al., 2018). In addition to the effect of these substances on plant growth and development, their positive effect on plant protection in stressful conditions has also been confirmed (Desta & Amare, 2021). Studies have shown that milk thistle and other plants react to the use of different plant growth regulators. For example, the use of Mepiquat chloride and thidiazuron in this plant has increased the seed yield and silymarin accumulation in the plant (Geneva et al., 2008).

The use of salicylic acid and hydrogen peroxide through foliar spraying and priming had a significant impact on improving the morphological characteristics, pigment content, and secondary metabolites of the marigold plant under cadmium toxicity conditions (Nizar et al., 2022). In a second study, the phytosanitary performance of milk thistle during drought stress has been improved by foliate spraying with salbutic acid (Ghassemi‐Golezani et al., 2016). The foliar spraying of *Silybum marianum* L. with salicylic acid at a concentration of 1 mM resulted in a significant increase in seed yield and oil content under drought conditions (Estaji & Niknam, 2020). Improvement in plant growth indices under drought stress conditions has been reported following the use of brassinosteroids in *Chrispora bungeana* (Li et al., 2012), *Capsicum annuum* (Hu et al., 2013), and *Cucumis sativus* (Yuan et al., 2012). Foliar spraying of *Echinacea purpurea* L. with salicylic acid and spermine under drought stress conditions has resulted in an increase in flavonoid concentrations and an improvement in growth indices (Darvizheh, Darvizheh, & Abbaszadeh, 2019; Darvizheh, Zahedi, et al., 2019). In another study, the use of salicylic acid (150 mg per liter) and spermine (70 mg L^−1^) in the plant *Echinacea purpurea* L. under drought stress conditions (irrigation after 60% depletion of soil) resulted in the highest recorded yield of cichoric acid, echinacoside, chlorogenic acid, caftaric acid, and cynarin in the plant's roots (Darvizheh, Darvizheh, & Abbaszadeh, 2019; Darvizheh, Zahedi, et al., 2019). Foliar spraying of *Carthamus tinctorius* L. with a foliar application of putrescine (60 micromolar) + spermine (40 μM) was effective in reducing the detrimental effects of drought stress (Toupchi Khosrowshahi et al., 2020).

The study of flavonoids of this plant was conducted in Baker & Schrall 1977. The analysis of flavonoids using HPLC was also done by Titel and Wagner (1977). After that, other studies were conducted in relation to the chemical compounds present in this plant. The study of flavonoids in the flower of Iraqi *Silybum marianum* L. using high‐performance liquid chromatography‐HPLC showed that the amount of 119.7143, 307.4991, 137.6423, 252.98, 339.91, 378.32, 234.64, and 127.25 μg g^−1^, respectively, from Taxifolin, Silychristin, Silidianin, Silychristin B, Siliybinin A, Silybinin B, Isosilybinin A, and Isosilybinin B are present in the flower. In this study, it was explained that *Silybum marianum* flowers are a rich source of flavonoids with high therapeutic potential (Al‐Samarrai et al., 2022). The cultivation of milk thistle is increasing due to its therapeutic health benefits and properties. Although this plant belongs to the group of drought‐resistant plants, exposing the plant to long‐term drought stress affects its seed quality and yield. Therefore, this research was conducted with the aim of studying some plant growth regulators such as salicylic acid, spermidine, and brassinosteroid to increase the plant tolerance to drought stress in milk thistle.

## 2 MATERIALS AND METHODS

### 2.1 The place of research

The present study was conducted at the Research Institute of Agriculture, Institute of Forest and Rangelands, Karaj, Iran, in 2021–2022. This research farm is located 5 km southeast of Karaj city at latitude 35 degrees 48 min north and 51 min east and an altitude of 1320 m above sea level with 14 degrees Celsius average annual temperature and about 235 mm average annual rainfall.

### 2.2 Plant materials and experimental treatments

At first, the seeds were planted in plots of 3 × 4 m at a depth of 1–2 cm. The distance between the blocks was 2 m and the distance between the main and subplots was 3 and 1 m, respectively. Row spacing was 50 cm and the plant spacing on the row was 60 cm.

Irrigation tapes with emitters spaced 20 cm apart have been used for plant irrigation. Irrigation tape of 6 m length was attached to each row of flowers. At the beginning of the experiment, the discharge of each emitter (measured volumetrically) was determined to be 2.4 L/h. The soil moisture level at field capacity was determined based on a soil sample obtained from the field and the soil moisture characteristic curve plotted by the National Institute of Forests and Rangelands Research. Soil moisture in the plots of each treatment has been determined by sequential sampling from the field and using a gravimetric method to determine the irrigation timing for all drought stress treatments. When the soil moisture reached the specified percentage, irrigation was performed until the soil moisture reached field capacity. Therefore, the irrigation frequency varied among different drought stress treatments. For the purpose of maintaining soil moisture within the range of field capacity, irrigation was carried out every 2 days during the growth period in the control group. Table 1 shows the number of irrigation events and the volume of water used for each treatment. The seed harvest was performed in the first half of June (late May).

**TABLE 1:** Number and volume of irrigation application to each drought stress treatment.

Treatment	25% FC	50% FC	75% FC	Control
Number of irrigations	9	11	17	40
Volume of irrigation (m^3^ ha^−1^)	28,560.0	2566.6	2425.3	2666.7

Experimental treatments included four levels of irrigation including 90, 75, 50, and 25 %FC and spraying at seven levels (75 and 150 mg L^−1^ salicylic acid, 70 and 140 mg L^−1^ spermine, and 1 and 1.2 μM brassinosteroid and control (water)). The time of using foliar spraying was at least three times during the vegetative, budding, and flowering stages. Each treatment had three replications.

### 2.3 Malondialdehyde measurement

The amount of malondialdehyde was measured by the method of Hess. Membrane peroxidation was measured by the thiobarbituric acid (TBAT) test. In this test, the amount of malondialdehyde (MDA) was measured from fresh plant tissues, which after determining fresh weight in 20% trichloroacetic acid (TCA) was homogenized with 0.5% thiobarbituric acid which was incubated for 30 min in a warm water bath (95°C) in a refrigerant balloon. Then the resulting mixture was immediately cooled in the ice bath and centrifuged at 10000 g for 30 min. The absorbance of the supernatant was determined at 532 nm and the specific absorbance at 600 nm was subtracted from it. Finally, the concentration of MDA was expressed as mol. G‐1 F.W.

### 2.4 Determination of leaf phenolic compounds

The content of phenolic compounds in the plant was also measured using the Folin‐Ciocalteu reagent. At first, to prepare the plant extract, 1 g of dried plant samples was weighed and well‐grounded under the conditions of using liquid nitrogen. Then, 5 mL of methanol acid was added to the powdered sample. Afterward, 0.5 mL of the resulting extract was added to 2.5 mL of 0.2 normal Folin‐Ciocalteu reagent. After 5 min, 2 mL of 75 g/L sodium carbonate solution was added to it. After 2 h, the absorbance of the mixture was read at 760 nm by a spectrophotometer (T80 + UV/VIS; PG Instruments Ltd) against the blank. Gallic acid was used as a standard to draw the calibration curve. The number of phenolic compounds was reported based on the amount equivalent to mg of gallic acid per gram of extract (Slinkard and Singleton, 1977).

### 2.5 Leaf flavonoid content

An aluminum chloride reagent was used to measure the amount of flavonoid in the leaves. First, to prepare plant extract, 1 g of dried plant sample was weighed and ground under conditions of using liquid nitrogen. Then, 5 mL of methanol acid was added to the powdered sample. After that, 1.5 mL of methanol, 0.1 mL of 10% ammonium chloride solution in ethanol, 0.1 mL of 1 M potassium acetate, and 8.2 mL of distilled water were added to 0.5 mL of plant extract. The absorbance of the resulting solution, 30 min after storage at room temperature, was read at 415 nm using a spectrophotometer (T80 + UV/VIS; PG Instruments Ltd) against a blank. Quercetin was used as a standard to draw the calibration curve. The flavonoid content was reported based on the equivalent amount of mg of Quercetin per g of extract (Chang et al., 2002).

### 2.6 Seed yield

To determine the seed yield in each plot, ten plants were first selected from the center of each plot to eliminate edge effects. Then, the fruits were manually harvested from each plant.

### 2.7 Analysis of plant extracts using HPLC

To extract silymarin, the milk thistle fruit was completely powdered and added to 3 g of the resulting 10 cc ethyl ether powder. The resulting solution was shaken for 30 min. It should be noted that during this time, the resulting solution was heated at 50°C in hot water. After that, the upper phase (oil) is separated from the solution (Parry et al., 2006). After degreasing, 1 cc of acidic methanol was added to 0.5 g of the dried sample in a microtube and placed in the refrigerator for 24 h. After centrifuging the resulting solution, the upper phase was separated and then 20 μL of the pure extract was injected into the device (Bakhshi & Arakawa, 2006). For the quantitative analysis of the compounds in the plant extract, a high‐performance liquid chromatography device was used (Gunaratna & Zhang, 2003; Quercia et al., 1980). The device included a Binary pump (Binary HPLC pump, 1525), UV detector (water dual absorbance 2487) and Symmetry C18 column (4.6 × 150 mm) with a flow rate of 1 mL min^−1^ was used (Table 2). The mobile phase consisted of acetonitrile, water, methanol, and formic acid. The total time in each chromatograph was 30 min. The extract was analyzed at wavelengths of 254 and 280 nm. A Dura silymarin capsule containing 26.165 mg silybinin was used as standard (Produced by JARROW Company). Finally, the values of each compound were determined based on the standard silymarin curve.

**TABLE 2:** Program of solvent system in HPLC.

No.	Time (min)	Flow (mL min^−1^)	%A	%B	Curve
1	—	1.00	95.0	5.0	—
2	3.00	1.00	70.0	30.0	6
3	13.00	1.00	65.0	35.0	6
4	23.00	1.00	55.0	45.0	6
5	35.00	1.00	55.0	45.0	6

### 2.8 Statistical analysis

Statistical analysis was done using JMP‐8 software. The mean comparison of data was done using the LSD test at a 5% probability level.

## 3 RESULTS

### 3.1 Phenolic compounds of leaf

**FIGURE 1:**
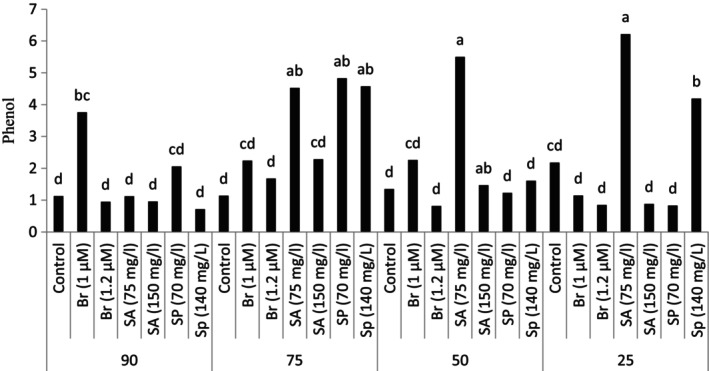
Phenolic compounds in plants grown in natural habitats and plants grown under experimental treatments. (Br: brassinosteroid, SA: salicylic acid, Sp: Spermidine).

The results of the research showed that plant exposure to drought stress conditions and using different plant growth regulators have been effective on the studied traits such as the amount of leaf phenolic compounds (Table 3). The study of the phenolic compounds in the leaves under stress conditions showed that the plant exposure to drought stress and soil moisture deficit causes to an increase in the phenolic compounds in the leaves compared to the control treatment (no stress). The highest amount of leaf phenol content was observed at the highest level of drought stress (25% of the field capacity) and foliar sprayed with 75 mg/L salicylic acid. In other stress levels, spraying the plant with 75 mg/L salicylic acid resulted in a significant increase in phenolic compounds in the plant. In addition, it was found that the foliar spraying with spermidine at 140 mg/L concentration increased the content of phenolic compounds in the plant compared to the control at the same level of stress. However, other experimental treatments were not significantly different from the control treatments at the same level of stress and control at 90% field capacity. In the condition of 75% of soil field capacity, spraying with spermidine in addition to salicylic acid (75 mg/L) significantly increased the phenolic compounds compared to the control treatment. It should be mentioned that the other experimental treatments were not significantly different from each other (Figure 1).

**TABLE 3:** Analysis of variance and mean square of traits studied in the research.

S.O.V	Df	Phenolic compounds	Malondialdehyde	Flavonoids	Seed yield
Drought	3	8.44^a^	1.03e‐6^b^	0.012^b^	98,410^b^
Block	2	1.04	8.96e‐8	0.0003	9840
Error a	6	0.96	8.18e‐8	0.00038	2775
PGRs	6	14.77^b^	1.32e‐6^b^	0.008^b^	18,785^b^
PGRs × Drought	18	5.52^b^	5.16e‐7^b^	0.0016^b^	14,342^b^
Error	48	1.23	1.2536e‐7	0.00012	1145

^a^ Significance at the 5% probability level.

^b^ Significant at the 1% probability level.

### 3.2 Leaf flavonoids

The flavonoid content of the leaves was influenced by different levels of dryness and plant growth regulators (*p* ≤ .01) (Table 3).

**FIGURE 2:**
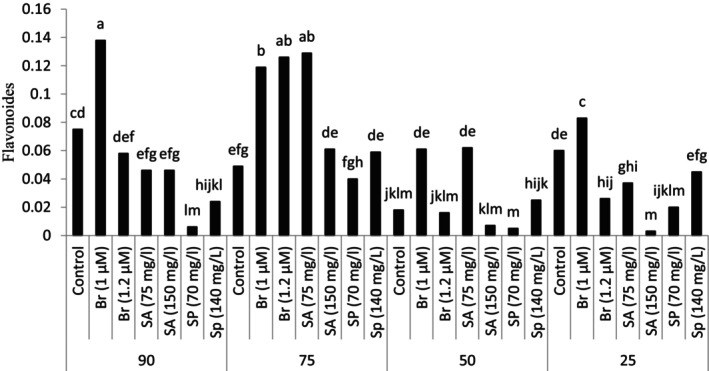
Leaf flavonoids in plants grown in natural habitats and plants grown under experimental treatments (Br: brassinosteroid, SA: salicylic acid, Sp: Spermidine).

The study of the number of flavonoids in the leaves showed that the highest amount of flavonoids was observed and recorded in the foliar spraying treatment of the plants grown under the conditions of 90% of the field capacity with 1 μM brassinosteroid. In addition, spraying the plant with brassinosteroid in different concentrations and salicylic acid at 75 mg/L caused a significant increase in the amount of flavonoids in the leaves compared to other experimental treatments. It should be noted that the amount of leaf flavonoids in other treatments was lower than the control (90% of field capacity and no spraying) (Figure 2).

### 3.3 Malondialdehyde

The research results demonstrated that the interaction of different levels of dryness and foliar application with a plant growth regulator had a significant effect on the malondialdehyde content (*p* ≤ .01) (Table 3).

**FIGURE 3:**
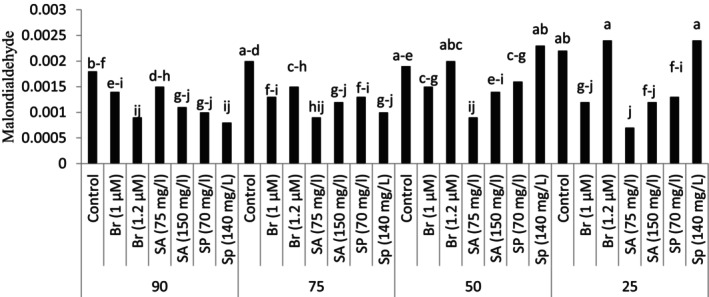
Malondialdehyde in plants grown in natural habitat and the plants grown under experimental treatments (brassinosteroid, SA: salicylic acid, Sp: Spermidine).

Foliar spray of plant growth regulators under stress conditions caused a significant change in the amount of leaf malondialdehyde content. In the conditions of 90% and 75% of the soil field capacity, foliar spray of plant growth regulators significantly reduced leaf malondialdehyde compared to the control treatment (no foliar spraying). Different results were observed in the conditions of 25% and 50% of the soil field capacity. In the plants grown under the conditions of 50% of the soil field capacity, only the use of 75 mg/L salicylic acid reduced the number of leaf flavonoids compared to the control treatment (no foliar spraying) at the same stress level. At the highest stress level, foliar spraying with salicylic acid, 1 μM brassinosteroid, and 70 mg/L spermidine significantly reduced leaf malondialdehyde in comparison with control treatment (no foliar spraying) at the same stress level (Figure 3).

### 3.4 Seed yield

**FIGURE 4:**
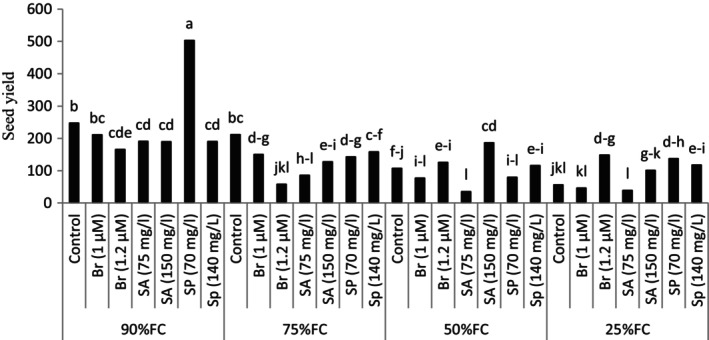
The effect of different levels of drought stress and plant growth regulator on seed yield.

The results of the research showed that the application of drought stress in the plant causes a significant decrease in the seed yield in the plant. However, foliar spraying with compounds improves the yield of seeds in the plant under stress conditions. The highest seed yield was observed in the treatment of FC 90% + spermine 70 mg per liter. In the condition of 50% of the agricultural capacity of the soil, spraying the plant with salicylic acid (150 mg/L) significantly increased the seed yield in the plant (nearly 75%) compared to the control treatment (no solution) at the same level of stress (Figure 4).

### 3.5 Quantitative analysis of silymarin by HPLC

**FIGURE 5:**
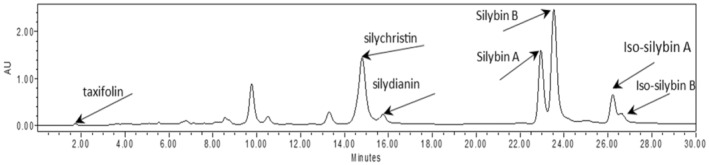
Chromatogram diagram of HPLC analysis of seed extract.

The results showed that the amount of taxifolin was affected by different research treatments in different conditions of the soil field capacity (Table 4). Taxifolin, silychristin, silydianin, silybinin A, silybinin B, iso‐silybinin A, and iso‐silybinin B compounds were identified in the analysis of the seed extract (Figure 5). The highest amount of Taxifolin was observed in the seeds of plants treated with 1 μM brassinosteroid in the moisture condition of 75% of the soil field capacity. The amount of Taxifolin in this treatment increased by 159% compared to the control treatment.

**TABLE 4:** Analysis of variance, mean square seed yield, and quantitative analysis of silymarin.

S.O.V	Df	Seed yield	Taxifolin	Silychristin	Silydianin	Silybin A	Silybin B	Iso‐silybin A	Iso‐silybin B
Drought	3	98,410^a^	27.8	11,468^a^	229,490	2.13e^+7**^	6,840,412^ns^	6,449,780^a^	359,301^a^
Block	2	9840	4.13^ns^	6.91^b^	111.6^ns^	278	174^ns^	4.77^ns^	11,706^ns^
Error a	6	2775	1.13	0.46	114.1	316	109	1.23	13,337^ns^
PGRs	6	18,785^a^	71.1^a^	47,062^a^	597,786^a^	1.66e^+7**^	4.16e+7^ns^	5,429,251^a^	180,086^a^
PGRs × Drought	18	14,342^a^	92.5^a^	45,852^a^	413,341^a^	2.26e^+7**^	2.69e+7^ns^	6,057,771^a^	366,329^a^
Error	48	1145	0.63	0.73	119	427	27,541	0.92	12,925

Abbreviation: ns, non‐significant.

^a^ Significant at the 1% probability level.

^b^ Significance at the 5% probability level.

In addition, the use of 70 mg/L spermidine also significantly increased the amount of this substance in the condition of 75% of the soil field capacity. In the condition of 50% of the soil field capacity, foliar spraying of brassinosteroid and 75 mg/L SA increased the amount of taxifolin compared to the control treatment by more than 147%. However, foliar spraying of the plants at 25% of the soil field capacity had no significant effect on the amount of this substance (Table 5).

**TABLE 5:** The study of the compounds in the seed extract of *Silybum marianum* (L.) Gaertn under soil moisture stress conditions and the use of the plant growth regulators.

FC (%)	Treatment	Taxifolin (μg)	Silychristin (μg)	Silydianin (μg)	Silybin A (μg)	Silybin B (μg)	Iso‐silybin A (μg)	Iso‐silybin B (μg)
90	Control	8.31efg	258n	209jk	5022k	19.16p	123mn	766e‐i
	Br (1 μM)	8.34efg	218q	245i	4422n	6092l	122no	818s‐h
	Br (1.2 μM)	6.37ijk	217q	278h	5522n	6426k	121o	917d‐h
	SA (75 mg/L)	4.52mno	320f	164op	843s	8102e	142j	664h‐k
	SA (150 mg/L)	18.8b	265L	219j	5369h	5374m	130L	609i‐l
	SP (70 mg/L)	8.6def	338d	176mno	670t	9150c	16.5t	715g‐k
	Sp (140 mg/L)	4.20no	177s	88.3r	6691d	6560jk	29.4s	1027abc
75	Control	0.00p	0.00x	0.00s	0.00w	0.00p	0.00u	0.00m
	Br (1 μM)	21.3a	298h	365f	5946g	8196e	0.68u	764e‐i
	Br (1.2 μM)	7.04ghi	281i	321g	5341h	7465g	2395b	946a‐e
	SA (75 mg/L)	5.44k‐n	226p	112q	675t	6149l	102r	474l
	SA (150 mg/L)	5.84i‐l	304g	483e	545u	7387gh	145i	902a‐f
	SP (70 mg/L)	20.59a	271k	1684a	1104r	7824f	132k	674h‐k
	Sp (140 mg/L)	4.74l‐o	275j	187lmn	5066j	7073i	124m	607i‐l
50	Control	6.82hij	198r	253i	3591o	4662n	1007c	572jkl
	Br (1 μM)	9.84d	352c	348f	6952c	47.4p	171g	1063ab
	Br (1.2 μM)	7.91e‐h	44.6v	1217b	1217q	7284ghi	124m	598i‐l
	SA (75 mg/L)	9.13de	81.7u	81.6r	182v	5334m	7287a	122m
	SA (150 mg/L)	5.26k‐n	438a	242i	8622a	11,186b	198d	887b‐g
	SP (70 mg/L)	3.72o	261m	251i	5139i	7164hi	121o	562kl
	Sp (140 mg/L)	4.89l‐o	177s	194kl	2635p	4088o	117p	1076a
25	Control	12.5c	281i	514d	5075j	6794j	133k	958a‐d
	Br (1 μM)	5.68j‐m	244o	117q	4855m	6749j	112q	6.04m
	Br (1.2 μM)	6.26ijk	84.0t	1088c	4934l	6793j	187e	0.00m
	SA (75 mg/L)	7.70fgh	33.2w	170nop	6195f	8477d	177f	751f‐j
	SA (150 mg/L)	0.00p	0.00x	0.00x	0.00w	0.00p	0.00u	0.00m
	SP (70 mg/L)	12.25c	429b	251i	8527b	11,532a	197d	701g‐k
	Sp (140 mg/L)	5.64j‐m	336e	190lm	6393e	8720d	152h	844c‐h

*Note*: The same letter in each column had no significance based on LSD (*p* ≤ .05) (brassinosteroid, SA: salicylic acid, Sp: Spermidine).

Foliar spraying of the plant using different plant growth regulators resulted in a change in the amount of Silychristin in the plant seed. Thus, the highest amount of this substance in the conditions of 90%, 75%, 50% and 25% of the soil field capacity were observed, respectively, in the treatments of salicylic acid 75 mg/L (319.57 μg), salicylic acid 150 mg/L (304.34 μg), salicylic acid 150 mg/L (439.41 μg), and spermidine 70 mg/L (429.98 μg) (Table 5).

The application of drought stress in the plant resulted in an increase in Silydianin concentration in the seeds. Specifically, in the treatment without foliar application at 50% and 25% of field capacity, compared to the control treatment (no foliar application +90% field capacity), Silydianin concentration increased by 21% and 146%, respectively. Furthermore, foliar application of the plant with a plant growth regulator in these conditions also led to an increase in Silydianin concentration in the seeds.

The highest amount of Silydianin was observed in the foliar spraying treatment of the plant using 70 mg L^−1^ spermidine in conditions of 75% of the soil field capacity (1684.04 μg). In this treatment, Silydianin content increased by more than 7‐fold compared to the control treatment. In addition, foliar spraying of the plant using brassinosteroid also increased the amount of Silydianin at different levels of drought stress compared to the control treatment at each level of stress (Table 5).

The results showed that the use of spermidine at a concentration of 140 mg/L in the condition of 90% of the soil field capacity increased the amount of silybin A by 33.2% compared to the control treatment (no spraying). In the condition of 75% of the soil field capacity, foliar spraying of the plant using 1 μM brassinosteroid increased the amount of silybin A by 18.3% compared to the control treatment in the condition of 90% of the soil field capacity. The highest amount of silybin A was obtained in the condition of 50% of the soil field capacity and plant spraying using 150 mg/L salicylic acid (8622.92 μg). At the highest level of stress, foliar spraying of the plant using spermidine and salicylic acid 75 mg/L increased the amount of silybin A compared to the control treatment at both levels of 90% and 25% of the soil field capacity (Table 5).

As a result of the application of drought stress, the concentration of silybinin B in the plant increased. In addition, an increase in the concentration of this compound at different soil moisture levels was also caused by the foliar application of the plant with a plant growth regulator. In all stress levels, foliar spraying of the plants with spermidine, salicylic acid, and brassinosteroid resulted in a significant increase in the amount of silybin B compared to the control treatment (no foliar spraying in the condition of 90% field capacity). The highest concentration of silybin B in the seed was observed under the soil moisture condition of 25% field capacity and foliar application of spermidine (70 mg/L) (Table 5).

The results showed that the use of salicylic acid at a concentration of 75 mg/L caused an increase of 15.4% in the amount of iso‐silybinin A compared to the control treatment (no foliar spraying) in the condition of 90% of the soil field capacity. Exposure to the plated water stress increased the amount of iso‐silybinin A in the plant. In addition, the plant treatment with 1.2 μM brassinosteroid and 150 mg/L salicylic acid at 50% and 75% of the soil field capacity increased the amount of this compound in the plant.

So, the highest concentration of iso‐silybin A was observed in the treatment of 50 mg/L salicylic acid +50% FC (Table 4).

The application of drought stress and foliar application of the plant with a plant growth regulator had an effect on the concentration of iso‐silybin B. Foliar application of the plant with spermidine at a concentration of 140 mg/L under 50% field capacity moisture condition increased the concentration of this compound by more than 40% compared to the control treatment (no foliar application +90% field capacity) (Table 5).

## 4 DISCUSSION

In recent years, various management programs have been implemented to reduce the effects of drought stress on plants with the aim of improving plant growth. External application of phytohormones such as salicylic acid is one of the most widely used strategies (Brito et al., 2019; Osama et al., 2019). SA is effective in biochemical and physiological processes (Estaji & Niknam, 2020). SA improves root growth (Brito et al., 2019) and prevents the reduction of auxin and cytokinin levels (Shakirova et al., 2003) in the plant. In addition, cell length and cell differentiation (Miura et al., 2009), ion absorption (Batista et al., 2019), and the plant antioxidant capacity are also affected by salicylic acid (Osama et al., 2019). In addition, SA improves enzymatic and photosynthetic activity and maintains the balance between ROS production and scavenging (Batista et al., 2019; Brito et al., 2019). SA is effective in reducing the negative effects of drought stress by improving the plant water relations and the accumulation of different osmolytes (Estaji & Niknam, 2020). In this study, foliar spraying of the plants with 75 M SA increased the amount of phenolic compounds in conditions of 25%, 50% and 75% of the soil field capacity. According to the results of this study, the increase in the level of phenolic compounds in the plant under drought stress conditions at the same time as the use of SA has been confirmed in other studies (Khan et al., 2015; Osama et al., 2019). In addition, the flavonoid compounds in the leaves increased under the foliar spraying of 75 M salicylic acid in the conditions of 50% and 75% of the soil's field capacity. One of the non‐enzymatic defense mechanisms in the plant is the production of phenolic compounds. These compounds are known as biochemical markers against environmental stress (Boudet, 2007) and play a role in increasing plant resistance to environmental stress, including drought stress by reducing ROS production (Mayer & Harel, 1991). Phenolic compounds as essential antioxidants in plants against the negative effects of oxidative stress play an important and decisive role in reducing or inhibiting lipid peroxidation, reducing free radicals, and eliminating reactive oxygen species (Ksouri et al., 2007). The increase in the number of phenolic compounds in the conditions of using SA in the plants is probably related to the increase in the activity of phenylalanine ammonia‐lyase as one of the enzymes involved in the production of related phenolic compounds (Chaman et al., 2003). Therefore, increasing the specific mRNA transcription of the phenylalanine ammonia‐lyase enzyme increased the activity of this enzyme and the biosynthesis of phenolic compounds. Then the accumulation of these compounds reduced the oxidative effects in the plant (Darvizheh, Darvizheh, & Abbaszadeh, 2019; Darvizheh, Zahedi, et al., 2019). On the other hand, the increase of other compounds under stress conditions such as sucrose and soluble sugar in the plant is effective in the accumulation of secondary compounds such as phenolic compounds in the plant (Bolouri‐Moghaddam et al., 2010; Ghasemzadeh et al., 2010). It has been explained that this tolerance to drought stress has a positive correlation with the accumulation of osmotic compounds in the plant (Hoekstra & Buitink, 2001). The improvement of tolerance to drought stress has been confirmed in other studies conducted on SA (El‐Esawi et al., 2017; Estaji & Niknam, 2020; Loutfy et al., 2012; Pacheco et al., 2013). Plant exposure to drought stress conditions results in the production of ROSs (reactive oxygen species) and then causes the peroxidation of lipids (Sairam et al., 2000). Malondialdehyde is produced as a result of the peroxidation of polyunsaturated fatty acids in phospholipids (Hessini et al., 2009; Kumari, Avtar, et al., 2018). Therefore, this compound is used as an indicator of membrane damage and lipid peroxidation (Kumari, Avtar, et al., 2018). In this research, spraying the plant using salicylic acid at different stress levels significantly reduced the amount of Malondialdehyde in the plant. In addition, foliar spraying of the plant using 1 μM brassinosteroid and 70 mg/L spermidine also had a positive effect in reducing the amount of Malondialdehyde in the plant at different levels of drought stress. Brassinosteroids play a role in cell strengthening by stimulating growth and creating defensive conditions (Sun et al., 2010). When the plants are exposed to stress conditions, brassinosteroids act as a secondary messenger, inducing the antioxidant defense system and destroying the reactive oxygen species (Mazorra et al., 2011) by regulating the activity of antioxidant enzymes present in cells (Ashraf et al., 2010), and play a role in preventing plasma membrane peroxidation (Mazorra et al., 2011). In addition, brassinosteroids increase resistance in plants by stimulating the expression of genes involved in defense, regulation, antioxidant responses, and production of high levels of H_2_O_2_ (Xia et al., 2009). It has been explained that spermidine as a plant growth regulator plays a role in the production of metabolites from different osmotic active substances (Alcazar et al., 2020; Ebeed et al., 2017). The use of spermidine and the accumulation of osmotic compounds in the plant increased the ability of plants to respond to drought strands to maintain balance in the plant and protect membranes and macromolecules. Spermidine probably plays a role in controlling redox homeostasis and maintaining the normal conditions of cell metabolic processes (Li et al., 2015; Sood & Nagar, 2003). In addition, the positive effect of spermidine on the accumulation of osmotic protectants has been confirmed to protect the plant against oxidative stress damage (Ebeed et al., 2017). The improvement of plant growth conditions under drought stress with the use of spermidine in the plants has also been observed in other studies (Gholizadeh et al., 2022; Tian et al., 2022).

The milk thistle plant has acceptable economic and medicinal value (Ram et al., 2005). On the other hand, this plant is easily adapted to different habitats. Therefore, by studying the compounds present in different ecotypes, it is possible to have the best choice for domestication in these plants (Radjabian, 2008). The amount of silymarin in the plant and its components depends on various factors such as the depth of planting, harvesting, and post‐harvest treatments and different parts of the plant. In addition, environmental factors such as rainfall, altitude, soil texture, and plant phenological stage are also effective on the plant silymarin content (Karkanis et al., 2011; Shokrpour et al., 2007). Previous studies have shown that the amount of silymarin in seed varies from 0.77 to 1.37 g per 100 g (Çağdaş et al., 2011). The study of the amount of silymarin in seeds harvested in Iran has shown that the amount of silymarin content varies from 23.98% to 45.46% (Radjabian, 2008). In a study conducted by Al‐Samarrai et al. (2022) in Iraq, it was found that *Silybum marianum* L. flowers contained Taxifolin (119.71 μg/g), Silychristin A (307.49 μg/g), Silidianin (137.64 μg/g), Silychristin B (252.93 μg/g), Silybin A (339.91 μg/g), Silybin B (378.32 μg/g), Isosilybin A (234.64 μg/g), and Isosilybin B (127.25 μg/g). The study of the amount of silymarin in four wild genotypes of *Silybum marianum* L. Gaertner in Syria showed that the geographical location of the place where the plant grows affects the amount of silymarin produced in the plant. Therefore, the total amount of silymarin in seeds varied from 0.54% to 2.91%. The highest concentration of silymarin was observed in seeds collected from Damascus and the lowest in Homs (Tayoub et al., 2018). It has been proven that chalcone synthase enzyme plays an important role in the silybin synthesis pathway, this role is in the addition of three malonyl‐CoA units to 4‐hydroxyvitamin CoA to produce naringenin as a precursor to *taxifolin*. Different developmental stages of the plant and the environmental stimuli are effective on the enzyme and mRNA levels of chalcone synthetase. This issue leads to changes in the final amount of compounds (Schmid et al., 1990). Spraying the plant with salicylic acid, brassinosteroid, and spermidine was effective in increasing the components of silymarin. In a study conducted by Yadegari et al. (2021), it was found that the inoculation of the plant with mycorrhiza increased the amount of flavonolignans in the plant (Yadegari et al., 2021). Secondary metabolites in plants are affected by the plant genotype and environmental conditions (Kutchan, 2001). Therefore, choosing the appropriate genotype and managing the environmental growth conditions should be considered (Zhang et al., 2019). In this research, it was also found that the cultivation of plants in the field was effective in the amount of compounds in silymarin. In addition, by the results of this study, it has been explained that plant exposure to drought stress conditions increases the compounds in silymarin. The amount of silybin A and silybin B increased by 24% and 26%, respectively, in the condition of water stress in the plant (Ghanbari Moheb Seraj et al., 2022). The change of secondary metabolites occurs under the influence of drought stress in the plant, which induces plant growth induction by carbon fixation during the photosynthesis process, which aims to increase the related secondary metabolites. The production of these compounds in plants is done with the aim of preventing cell oxidation (Pirzad et al., 2006; Turtola et al., 2003).

## 5 CONCLUSION

Secondary metabolites and silymarin content in milk thistle plants are important factors in the cultivation of this medicinal plant. The importance and economic value of this medicinal plant is influenced by the compounds of silymarin. In addition to plant genotype, environmental conditions are one of the most influential factors in the production of secondary metabolites in plants. In this research, it was found that the cultivation of native plants in field conditions and the use of drought stress increased the number of secondary metabolites and the number of silymarin constituents in the plant. In addition, foliar spraying of plants using plant growth regulators such as salicylic acid had a significant effect on increasing the silymarin components and the production of secondary metabolites in the plant. Also, brassinosteroids and spermidine are suitable options in some environmental conditions to improve plant growth conditions under drought‐stress conditions.

## AUTHOR CONTRIBUTIONS

**Sahar Fanai:** Data curation (equal); formal analysis (equal); methodology (equal); validation (equal); writing – review and editing (equal). **Davood Bakhshi:** Conceptualization (equal); investigation (equal); supervision (equal); writing – original draft (equal). **Bohloul Abbaszadeh:** Conceptualization (equal); project administration (equal); supervision (equal); visualization (equal).

## CONFLICT OF INTEREST STATEMENT

The authors declare no conflict of interest.

## ETHICS STATEMENT

Our research did not contain any animal experiments or human subjects.

## Data Availability

The data that support the findings of this study are available from the corresponding author upon reasonable request.
